# Draft Genome Sequence of *Lactobacillus oryzae* Strain SG293^T^

**DOI:** 10.1128/genomeA.00861-14

**Published:** 2014-08-28

**Authors:** Yasuhiro Tanizawa, Takatomo Fujisawa, Takako Mochizuki, Eli Kaminuma, Yasukazu Nakamura, Masanori Tohno

**Affiliations:** a Department of Computational Biology, Graduate School of Frontier Sciences, the University of Tokyo, Chiba, Japan; b Genome Informatics Laboratory, National Institute of Genetics, Mishima, Japan; c National Agriculture and Food Research Organization, National Institute of Livestock and Grassland Science, Nasushiobara, Japan

## Abstract

We report the 1.86-Mb draft genome and annotation of *Lactobacillus oryzae* SG293^T^ isolated from fermented rice grains. This genome information may provide further insights into the mechanisms underlying the fermentation of rice grains.

## GENOME ANNOUNCEMENT

In the livestock industry, lactic acid bacteria (LAB) are widely used as inoculants for silage fermentation. Recently, for livestock feed, rice grains have gained attention as an alternative to corn (1). During an investigation of LAB from fermented rice grains, *Lactobacillus oryzae*, a novel species of the genus *Lactobacillus*, was isolated (2). It may be associated with adequate fermentation of rice grain silage and has the potential for biotechnological application, such as an effective inoculant for rice grains. Here we report the draft genome sequence of strain SG293, which is a type strain of *L. oryzae*.

The genomic DNA of SG293^T^ was extracted and purified from a culture grown in De Man, Rogosa, and Sharpe broth (Difco) using QIAGEN Genomic tips (Qiagen). The whole genome was sequenced using the 250-bp pair-end Illumina MiSeq system (Filgen, Japan), yielding a total of 8,545,944 reads, which corresponds to approximately 1,100-fold coverage. Reads that contained adaptor sequences were filtered using the cutadapt software (3). *De novo* assembly was performed using Platanus version 1.2.1 (4). Contigs shorter than 300 bp were eliminated.

The draft genome was annotated by the Microbial Genome Annotation Pipeline (MiGAP) online annotation server (5), which combines prediction of open reading frames by MetaGeneAnnotator 1.0 (6), tRNA prediction by tRNAscan-SE 1.23, rRNA prediction by RNAmmer 1.2, and homology searches against the RefSeq, TrEMBL, and Clusters of Orthologous Groups (COGs) protein databases. The KEGG Automatic Annotation Server (KAAS) (7) was also used to search for metabolic pathways. In addition to these automated annotations, protein sequences were queried against the NCBI-nr and Swiss-Prot databases using BLASTp, searched against signature/domain databases using InterProScan, and part of the annotation was curated manually. Clustered regularly interspaced short palindromic repeats (CRISPR) loci were searched using the CRISPRfinder server (8). The annotated genome was submitted to the GenomeRefine web service (http://genome.annotation.jp/genomerefine/), which assists with the refinement of the annotation and registration at the International Nucleotide Sequence Database Collaboration (INSDC) through DDBJ.

The draft genome sequence of *L. oryzae* SG293^T^ consists of 93 contigs and includes 1,860,394 bp with an overall G+C content of 43%. In the genome, 1,891 protein-coding sequences (CDSs) as well as 59 copies of tRNA and six copies of rRNA were predicted. Approximately 73% of the CDSs were assigned to specific COGs and 53% were assigned a KEGG orthology (KO) number. All enzymes required for the phosphoketolase pathway are encoded in the genome, whereas some genes involved in the Embden–Meyerhof–Parnas pathway are absent, supporting the classification of this strain as an obligate heterofermentative LAB. One CRISPR locus was detected in the genome, which contained three CRISPR-associated proteins (LOSG293_470040–470060) and 47 spacer sequences separated by 36-bp direct repeats. This genomic information may provide further insights into the mechanisms underlying the fermentation of rice grains.

### Nucleotide sequence accession numbers.

This whole-genome shotgun project has been deposited in DDBJ/ENA/GenBank under the accession no. BBJM00000000. The version described in this paper is the first version, BBJM01000000.
